# Autoantibodies against the Catalytic Domain of BRAF Are Not Specific Serum Markers for Rheumatoid Arthritis

**DOI:** 10.1371/journal.pone.0028975

**Published:** 2011-12-12

**Authors:** Wenli Li, Wei Wang, Shipeng Sun, Yu Sun, Yang Pan, Lunan Wang, Rui Zhang, Kuo Zhang, Jinming Li

**Affiliations:** 1 National Center for Clinical Laboratories, Beijing Hospital, Beijing, People's Republic of China; 2 Graduate School, Peking Union Medical College, Chinese Academy of Medical Sciences, Beijing, People's Republic of China; University of California San Francisco, United States of America

## Abstract

**Background:**

Autoantibodies to the catalytic domain of v-raf murine sarcoma viral oncogene homologue B1 (BRAF) have been recently identified as a new family of autoantibodies involved in rheumatoid arthritis (RA). The objective of this study was to determine antibody responses to the catalytic domain of BRAF in RA and other autoimmune diseases. The association between RA-related clinical indices and these antibodies was also assessed.

**Methodology/Principal Findings:**

The presence of autoantibodies to the catalytic domain of BRAF (anti-BRAF) or to peptide P25 (amino acids 656–675 of the catalytic domain of BRAF; anti-P25) was determined in serum samples from patients with RA, primary Sjögren's syndrome (pSS), systemic lupus erythematosus (SLE), and healthy controls by using indirect enzyme-linked immunosorbent assays (ELISAs) based on the recombinant catalytic domain of BRAF or a synthesized peptide, respectively. Associations of anti-BRAF or anti-P25 with disease variables of RA patients were also evaluated. Our results show that the BRAF-specific antibodies anti-BRAF and anti-P25 are equally present in RA, pSS, and SLE patients. However, the erythrocyte sedimentation rate (ESR) used to detect inflammation was significantly different between patients with and without BRAF-specific antibodies. The anti-BRAF-positive patients were found to have prolonged disease, and active disease occurred more frequently in anti-P25-positive patients than in anti-P25-negative patients. A weak but significant correlation between anti-P25 levels and ESRs was observed (r = 0.319, p = 0.004).

**Conclusions/Significance:**

The antibody response against the catalytic domain of BRAF is not specific for RA, but the higher titers of BRAF-specific antibodies may be associated with increased inflammation in RA.

## Introduction

Autoimmune diseases occur when the body's immune system attacks self-antigens. This induces prolonged inflammation and subsequent tissue destruction. Rheumatoid arthritis (RA), a common systemic autoimmune disease of unknown etiology, is characterized by chronically inflamed synovial joints and subsequent destruction of cartilage and bones. Despite decades of research, the pathogenesis of RA is still unresolved. One of the hallmarks of RA is the presence of a broad spectrum of autoantibodies against aberrantly expressed autoantigens. The discovery of autoantibodies to citrullinated proteins such as fibrin and vimentin in patients with RA was one of the most important findings in rheumatology research [1]. Advances in protein array technologies have enabled large-scale analysis of proteins to identify significant biomarkers that contribute to disease pathogenesis. A recently published paper describing 8,268 protein arrays using RA sera indicates that the catalytic domain of v-raf murine sarcoma viral oncogene homologue B1 (BRAF) is a new autoantigen for RA [2].

BRAF is a serine-threonine kinase involved in the mitogen-activated protein kinase (MAPK) pathways that regulate cell survival, proliferation, differentiation, cytokine generation, and metalloproteinase production [3]. BRAF somatic missense mutations are reported in 66% of malignant melanomas and at a lower frequency in a wide range of other human cancers [4]. A mutated BRAF gene with a single amino acid substitution (BRAF V600E) results in higher kinase activity. Thus, the resulting BRAF protein, which has protective activity against Raf kinase inhibitors, has been considered as a potential target for tumor therapy [5]. On the other hand, the MAPK pathways are implicated in the pathogenesis of certain inflammatory autoimmune diseases such as RA via their regulatory effects on the production of cytokines or metalloproteinases [6]–[9]. Recent data show that serum antibodies to the catalytic domain of BRAF (anti-BRAF) can activate BRAF *in vitro*. This indicates that anti-BRAF may play a role in inflammation in RA through activation of the MAPK pathway [10]. The results of peptide array analysis indicate that the antibody response to P25 (amino acids 656–675 of the catalytic domain of BRAF) is specific to RA. However, antibodies to peptide P25 (anti-P25) were defined as specific markers for RA, based on comparison to small patient cohorts with ankylosing spondylitis (AS) and psoriasis arthritis (PsA), rather than to patients with autoimmune disorders. In the present study, we determined the antibody responses to the catalytic domain of wild-type BRAF and peptide P25 in Chinese patients with RA, primary Sjögren's syndrome (pSS), and systemic lupus erythematosus (SLE) by indirect enzyme-linked immunosorbent assays (ELISAs) and investigated the possible associations between these antibodies and the disease indicators of RA.

## Materials and Methods

### Ethics statement

Written informed consent was not obtained because of the nature of the study design, which utilized serum samples taken after routine tests. All subjects recruited in this study were informed of the nature of the project and verbal informed consent was obtained from each patient, This was recorded by the physician who explained the study procedure. The study protocol and verbal consent document were approved by the Ethics Committee of the National Center for Clinical Laboratories, where the study was performed.

### DNA constructs

The DNA segment corresponding to the catalytic domain of wild-type BRAF (amino acids 416–766) was generated by PCR using specific primers carrying restriction sites. The pEF-myc-BRAF plasmid containing full-length human BRAF cDNA, was kindly provided by Dr. Richard Marais (Institute of Cancer Research, London, United Kingdom). Enzyme-restricted PCR products were ligated into the multiple cloning sites of the pET28b expression vector by T4 DNA ligase. The desired clones were confirmed by sequencing.

### Protein expression and purification

The recombinant plasmid carrying the catalytic domain of wild-type BRAF (pET28b-BRAF) was transformed into *Escherichia coli* BL-21(DE3). Further, a 6× His-tagged protein was expressed with induction by 0.1 mM isopropyl-β-D-thiogalactoside (IPTG) for 4 h at 37°C. Bacterial pellets from a total of 1 L of culture were resuspended in 10 mL lysis buffer (50 mM Tris-Cl, 100 mM NaCl, 5 mM EDTA, 1% NaN_3_, 0.5% Triton X-100, 5 mM DTT, pH 8.0). After the suspension was prepared, lysozyme (Sigma-Aldrich, St. Louis, MO, USA) was added to a final concentration of 0.2 mg/mL, followed by incubation at room temperature (RT) for 30 min. The cells were further disrupted by sonication on ice for 10 min (on for 5 s, off for 5 s). The homogenate was then centrifuged at 4°C for 30 min at 6000 *g*. The supernatant was discarded, and the inclusion bodies were collected. The collected precipitates were resuspended in 10 mL washing buffer (100 mM Tris-Cl, 5 mM EDTA, 5 mM DTT, 2 M urea, 2% Triton X-100, pH 8.0) and incubated at RT for 20 min. The inclusion bodies were then recovered by centrifugation at 4°C for 30 min at 8000 *g*. The above washing step was repeated twice, the inclusion bodies were dissolved in binding buffer (20 mM sodium phosphate, 0.5 M NaCl, 40 mM imidazole, 1.5% Triton X-100, 4 mM DTT, 6 M guanidine-HCl, pH 8.0), and the recombinant protein was further purified by affinity chromatography on a Ni-Sepharose Fast flow (FF) column (GE Healthcare, Uppsala, Sweden). The His-tagged protein was eluted with a linear concentration gradient of imidazole from 40 to 400 mM. The fractions containing the target protein were pooled, dialyzed to remove imidazole, and stored in the presence of 6 M guanidine-HCl at −20°C. The protein concentration was determined by a standard bicinchoninic (BCA) protein assay (Pierce, Rockford, USA). To evaluate the size and purity of the recombinant protein, samples were denatured in SDS loading buffer (25 mM Tris-HCl, pH 6.8, 5% β-mercaptoethanol, 2% SDS, 50% glycerol), separated on a 10% polyacrylamide gel, and stained with Coomassie blue.

### Serum samples

Serum samples were obtained from a previously described RA cohort that fulfilled the American College of Rheumatology (ACR) criteria for RA [11], [12] and included 101 patients in the final study. For comparison, samples from 250 subjects with other autoimmune diseases were tested, including samples obtained from 132 patients with pSS and samples obtained from 118 patients with SLE. Healthy controls (140) were also included to determine the cutoff value for positivity. Serum samples were stored at −80°C until analysis. The following data were collected from RA patients: gender, age, disease duration, rheumatoid factor (RF), anti-cyclic citrullinated peptide antibodies (anti-CCP), erythrocyte sedimentation rate (ESR), C-reactive protein (CRP), and disease status. Recent-onset RA was defined as RA with disease duration of less than 2 years. RF and CRP levels were determined by an immunonephelometric method. Values >7.9 mg/L for CRP and >20 IU/mL for RF were considered positive. Anti-CCP antibodies were assessed with a commercial ELISA kit (Immunoscan CCPlus, Euro-Diagnostica, Malmo, Sweden) according to the manufacturer's recommendations. The cutoff value for a positive reaction was set at 25 U/mL, as suggested by the manufacturer. The ESR was measured by Westergren's method; values ≤15 mm/h for men and ≤20 mm/h for women were considered normal. Active RA was defined as described previously [12]. The basic characteristics of the RA cohort are described in Table 1.

**Table 1:** Demographic data and disease indicators of 101 patients with RA.

	Number	Description
Females/Males	101	81/20
Age, years	101	47.3±13.8
Disease duration, years	97	5 (0.1–50)
Recent onset	35	1 (0.1–2)
Prolonged	62	8 (3–50)
RF	97	
RF-positive	83	82.2%
Anti-CCP(U/mL)	101	353 (16–5477)
Anti-CCP-positive	74	811 (25–5477)
ESR, mm/h	81	56±33
Normal	14	12±5
Elevated	67	66±28
CRP	62	
Elevated	25	40.3%
Disease status	101	
Active disease	47	46.5%

RF: rheumatoid factor; anti-CCP: anti-cyclic citrullinated peptide antibodies; ESR: erythrocyte sedimentation rate; CRP: C-reactive protein.

Categorical variables are given as %; normally distributed data are given in mean ± SD; other continuous variables are given in median (range).

### Detection of IgG anti-BRAF by ELISA

Specific antibodies to the recombinant catalytic domain of wild-type BRAF were identified in sera by an indirect ELISA. To conduct the assay, 100 µL of the recombinant catalytic domain of BRAF (2.5 µg/mL) was incubated in an ELISA plate (Nunc Maxisorp, Roskilde, Denmark) at 4°C overnight. Microwells were then washed with phosphate-buffered saline (PBS: 0.01 M, pH 7.4) with 0.05% Tween-20 (PBST). Unbound sites were blocked by incubation with 200 µL 20% newborn calf serum (NCS) in PBS at 37°C for 1.5 h. Sera were diluted 1∶200 in blocking buffer and aliquots of 100 µL were added to the wells. Wells coated with bovine serum albumin (BSA) were prepared for each sample, to assess non-specific binding. After incubation at 37°C for 1 h, plates were washed 3 times with PBST. Subsequently, the captured antibodies were detected by a horseradish peroxidase (HRP)-conjugated goat anti-human IgG (1∶10000) (Sigma), which was diluted with 20% NCS in PBST (100 µL/well). After incubation at 37°C for 30 min, wells were washed 5 times with PBST. Color was developed by application of 100 µL of tetramethylbenzidine (Sigma) at 37°C for 20 min. The reaction was stopped by addition of 0.5 M sulfuric acid, and the optical density at 450 nm (OD450), with 620 nm as the correction wavelength, was obtained using an ELISA plate reader (Labsystems, Finland).

Each sample was assayed in duplicate. A positive serum sample was included in each assay and used to correct for inter-assay variations. Results were expressed as arbitrary units (AU) calculated as ([OD_450_ of sample−OD_450_ of the non-specific binding of the sample]/[OD_450_ of the positive control−OD_450_ of the non-specific binding of the positive control])×100.

### Detection of IgG autoantibodies to P25 by ELISA

To test patient reactivity to peptide P25 (YSNINNRDQIIFMVGRGYLS, a peptide encompassing amino acids 656–675 of the catalytic domain of BRAF), an indirect ELISA for quantifying IgG autoantibodies to P25 was conducted. Serum samples from RA, pSS, and SLE patients were included in the assay. Eighty-nine of 140 healthy controls were also included to evaluate the cutoff value. To efficiently coat microwells with the peptide, BSA-conjugated P25, synthesized by the Chinese Peptide Company (Hangzhou, Zhejiang, China) via the solid-phase method, was used as an antigen. The purity of the conjugate was greater than 95%. Plates were coated overnight with BSA-P25 at a concentration of 5 µg/mL. After blocking unbound sites, the serum samples were diluted 1∶100 and incubated with the plates at RT for 1 h. Wells coated with BSA were prepared for each sample to determine non-specific binding. After washing, HRP-conjugated goat anti-human IgG was added and incubated at RT for 1 h. The plate was read at an OD of 450 nm, with 620 nm as the correction wavelength, using an ELISA plate reader.

Each sample was tested in duplicate. A positive serum sample was included in each assay and used to correct for inter-assay variations. Data was processed as described in the anti-BRAF ELISA procedure.

### Statistical analyses

Statistical analyses were performed using SPSS 13.0 for Windows. For normally distributed data, results are expressed as the mean and standard deviation (mean (SD)); differences between groups were assessed by *t*-tests. For data not distributed normally, results are expressed as the median (range); differences between groups were analyzed using the Mann-Whitney *U*-test and correlations were determined by computing Spearman rank correlation coefficients. Pearson's 2-tailed χ^2^ test or Fisher's exact test were used to compare proportions. P values<0.05 were considered statistically significant.

## Results

### Expression and purification of recombinant protein

**Figure 1:**
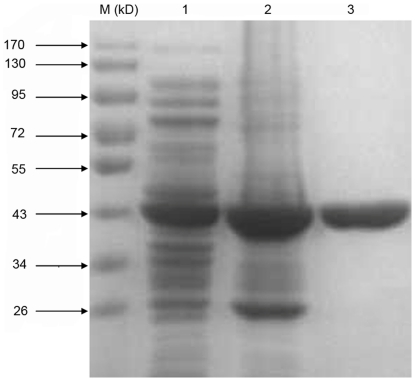
Analysis of the recombinant catalytic domain of BRAF by SDS-PAGE. Samples were separated by electrophoresis on polyacrylamide gels and stained with Coomassie blue. M: molecular mass marker proteins. Lane 1: BL21-(DE3) cells carrying pET28b-BRAF plasmid induced by 0.1 mM IPTG for 4 h at 37°C. Lane 2: inclusion bodies after extraction. Lane 3: 6× His-tagged proteins eluted with imidazole. The weight of the molecular mass markers is indicated on the left side of the figure.

The recombinant catalytic domain of wild-type BRAF was expressed from pET28b-BRAF-transformed bacteria under IPTG induction. The expressed protein was within insoluble inclusion bodies. To obtain pure antigens, a protocol for inclusion-body extraction followed by affinity chromatography was implemented. Following extraction, recombinant proteins were predominantly identified in collected precipitates, but remained contaminated with a small quantity of host proteins. For further purification, precipitates were solubilized in 6 M guanidine-HCl and purified using nickel affinity chromatography under denaturing conditions. The His-tagged recombinant proteins were eluted with a gradient of increasing imidazole concentration and were detected as a single protein band at a molecular weight of approximately 40 kD on a 10% SDS-PAGE gel (Figure 1). The protein concentration was 1.5 mg/mL as determined by BCA.

### Prevalence of antibody responses to BRAF in diseases and controls

**Figure 2:**
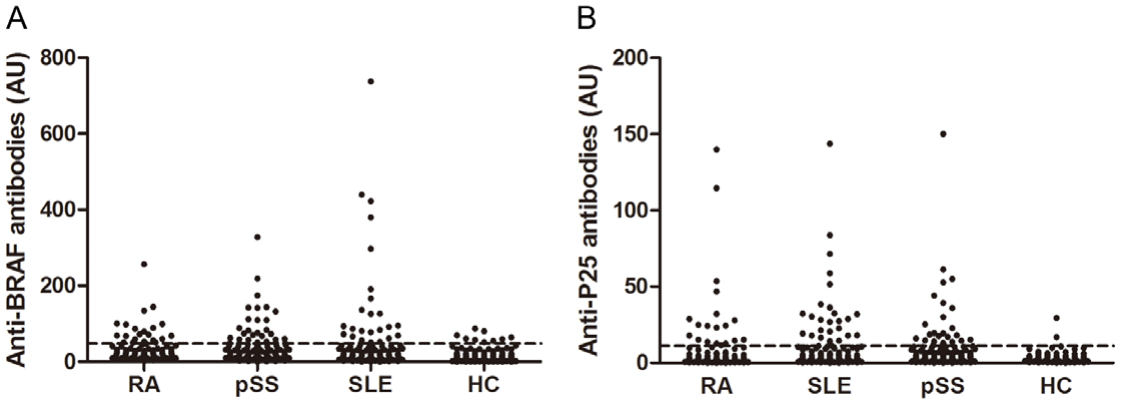
Distribution of BRAF-specific antibodies in diseases and controls. BRAF-specific antibodies were detected in patients with rheumatoid arthritis (RA, n = 101), primary Sjögren's syndrome (pSS, n = 132), systemic lupus erythematosus (SLE, n = 118), and healthy controls (HC, n = 140 for anti-BRAF and n = 89 for anti-P25) using indirect ELISAs based on the recombinant catalytic domain of BRAF (**A**) or a synthesized peptide (**B**). Antibody titers were expressed as arbitrary units (AU). The cutoff value for positivity was set as 2 SD above the mean AU of the healthy controls (dashed line).

The distribution of BRAF-specific antibodies in RA, pSS, SLE and healthy control patients is shown in Figure 2. The cutoff value for positivity was set as 2 SD above the mean AU of the healthy controls. The prevalence of anti-BRAF and anti-P25 is listed in Table 2. There was no significant difference in anti-BRAF or anti-P25 prevalence among RA, pSS, and SLE patients. However, the prevalence of BRAF specific antibodies was significantly higher in disease samples (RA, pSS, and SLE) than in the healthy controls (p = 0.001 for all). 8 serum samples of RA patients were identified as anti-P25 positive and anti-BRAF negative, whereas another 10 RA samples were identified as anti-P25 negative and anti-BRAF positive. A similar tendency was also observed among pSS and SLE patients.

**Table 2:** Prevalence of BRAF specific antibodies in the test samples.

Disease	anti-BRAF positive (%)	anti-P25 positive (%)	anti-BRAF positive & anti-p25 negative	anti-BRAF negative & anti-p25 positive
RA	21/101 (20.8)	19/101 (18.8)	10	8
SLE	24/118 (20.3)	25/118 (21.2)	9	10
pSS	27/135 (20.5)	24/132 (18.2)	12	9
HC	9/140 (6.4)	2/89 (2.2)	3	0

Since the anti-p25 was not test in all the patients and health controls, the results we list in the last two columns were from the participants that both anti-BRAF and anti-p25 were tested.

### Associations between BRAF-specific antibodies and disease indicators in RA patients

**Figure 3:**
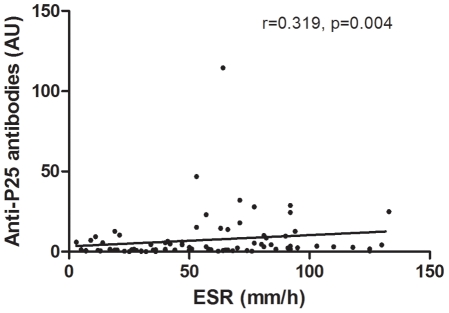
Correlation of anti-P25 antibodies with ESRs in RA patients. The correlation of anti-P25 antibodies and ESRs in 81 RA patients was assessed by Spearman rank correlation coefficients. The coefficient (r = 0.319, p = 0.004) suggests a weak but significant association between anti-P25 antibodies and ESR values.

Of the 101 RA patients, 21 (20.8%) and 19 (18.8%) were identified as positive for anti-BRAF and anti-P25, respectively. Patients with BRAF-specific antibodies had significantly higher ESRs than patients without these antibodies (p = 0.040 for anti-BRAF and p = 0.030 for anti-P25). Patients with prolonged disease had a significantly higher prevalence of anti-BRAF (18/62) than patients with recent-onset disease (2/35) (p = 0.006). Furthermore, active disease occurred more frequently in anti-P25-positive patients than in anti-P25-negative patients (p = 0.034). Comparisons of disease indicators between patients with and without BRAF-specific antibodies are shown in Table 3. A weak but significant correlation was found between anti-P25 antibodies and ESRs in the RA patients (r = 0.319, p = 0.004) (Figure 3).

**Table 3:** Comparisons of disease indicators between patients with and without BRAF-specific antibodies.

	Anti-BRAF catalytic domain		p	Anti-P25		p
	Positive	Negative		Positive	Negative	
Female (%)	85.7	78.8	0.685	81.3	86.3	0.604
Age (years)	51.5±12.7	46.2±14.0	0.116	49.6±11.6	46.7±14.3	0.429
Duration (years)	7.5 (0.3–30)	4.8 (0.1–50)	0.073	5.0 (0.2–14)	5.0 (0.1–50)	0.874
Recent onset (%)	10.0	42.9	0.006	22.2	39.2	0.175
RF-positive (%)	80.0	87.0	0.661	94.4	83.5	0.456
Anti-CCP (U/mL)	46 (17–2572)	367 (16–5477)	0.490	357 (17–3799)	338 (16–5477)	0.281
Positive (%)	66.7	75.0	0.443	84.2	70.7	0.232
ESR (mm/h)	69.3±31.6	52.0±32.2	0.040	71.8±26.3	53.0±33.2	0.030
Elevated (%)	90.0	80.3	0.499	92.9	80.6	0.444
CRP elevated	33.3	42.0	0.747	44.4	39.6	1.000
Active disease (%)	42.9	47.5	0.704	68.4	41.5	0.034

RF: rheumatoid factor; anti-CCP: anti-cyclic citrullinated peptide antibodies; ESR: erythrocyte sedimentation rate; CRP: C-reactive protein.

Categorical variables are given as %; normally distributed data are given in mean ± SD; other continuous variables are given in median (range). Recent onset disease is defined as disease duration of less than 2 years.

## Discussion

Autoantibodies to BRAF, in particular anti-P25 antibodies, have been recently identified as specific markers for RA. However, this suggestion is based on the evidence that anti-P25 is specifically detected in RA patients comparing with AS and PsA. In this report, we developed indirect ELISAs on the basis of the recombinant catalytic domain of BRAF or the synthesized peptide P25 and determined the prevalence of autoantibodies to BRAF in patients with RA, pSS, or SLE and in healthy controls. Associations between anti-BRAF or anti-P25 and disease variables were investigated in the RA cohort. Our results indicate that neither anti-BRAF nor anti-P25 autoantibodies are specific markers for RA. Nevertheless, the associations between anti-BRAF or anti-P25 and disease variables suggest potential involvement of these antibodies in inflammation in RA patients.

Protein arrays have been used to identify the catalytic domain of BRAF as a new autoantigen involved in RA [2]. Recently, Charpin et al. [10] further identified the peptide targets of anti-BRAF by using 40 overlapping 20-mers encompassing the entire catalytic domain of BRAF. It was shown that 1 peptide, P25 (amino acids 656–675), is specifically recognized by anti-BRAF from serum of RA patients [10]. In the present study, we detected the presence of anti-BRAF and anti-P25 in the serum of RA patients by developing indirect ELISAs on the basis of the recombinant catalytic domain of BRAF in its denatured form and a synthesized peptide P25, respectively. Recombinant proteins dissolved in denaturant have been successfully used to coat antigens in ELISAs. This ensures the validity of our assays for anti-BRAF [13]–[14]. We unexpectedly observed a considerable prevalence of anti-BRAF and anti-P25 in pSS patients and SLE patients. In the previous 2 studies investigating anti-BRAF in RA patients, the disease controls were AS patients and/or PsA patients, and cohorts used were relatively small [2], [10]. Thus, the involvement of autoantibodies to BRAF in other autoimmune diseases is still unclear. Here, we detected the presence of BRAF-specific antibodies in larger cohorts of patients with pSS and SLE. The prevalence of anti-BRAF (catalytic domain) or anti-P25 in these 3 diseases (RA, pSS, and SLE) is similar, This suggests that, to some extent, the production of autoantibodies to BRAF might be a common event in systemic autoimmune disorders. There is evidence that different subsets of autoantibodies have different cytokine requirements [15]. Thus, the indistinguishable prevalence of BRAF-specific antibodies among RA, pSS, and SLE patients raises the possibility that the cytokine environment in these diseases is beneficial for anti-BRAF or anti-P25 production. The repertoire of epitopes that elicit antibody responses to the catalytic domain of BRAF might include both linear and conformational forms. For the protein microarray, the catalytic domain of BRAF was adhered to the glass slide under native conditions. In contrast, in the peptide microarray, overlapping linear peptides of the catalytic domain were used as antigens [2], [10]. In our study, it is possible that both linear and conformed epitopes of the catalytic domain of BRAF were involved, as the process by which recombinant BRAF was diluted with coating buffer in denaturant may have caused refolding. Thus, some epitopes probably become inaccessible because of partial refolding or aggregation. This would lead to lower detection sensitivity for a specific peptide. This might account for some samples that were identified as anti-P25 positive but anti-BRAF negative. Furthermore, the difference in the final molar concentration of P25 adsorbed on the microwells between the 2 ELISAs is worthy of consideration.

Multiple signal transduction pathways have been carefully investigated in RA. For instance, NF-κB and MAPK pathways are attractive for intervention in light of their ability to regulate many genes involved in immune responses [16]–[17]. The enormous diversity of kinases that modulate transduction mechanisms suggests that complex and interrelated events are involved in inflammatory disease. The end results of these pathways may exert influences on the production of proteins such as cytokines and matrix metalloproteinases that are implicated in the pathogenesis of RA [18]–[20]. BRAF encodes a serine-threonine kinase downstream of RAS in the MAPK pathway and transduces regulatory signals from RAS through MAPK. Autoantibodies to the BRAF protein have been reported in melanoma patients and patients with RA [2], [10], [21]. Most recently, Charpin, et al. demonstrated that anti-BRAF may activate phosphorylation of MEK1 by using BRAF *in vitro*. This indicates possible involvement of BRAF autoantibodies in the inflammatory responses of RA [10]. Here, we observe a significant difference in ESRs between RA patients with BRAF-specific antibodies and those without these antibodies (p = 0.040 for anti-BRAF and p = 0.030 for anti-P25). Furthermore, a weak but significant correlation was identified between ESRs and anti-P25 antibody levels (r = 0.319, p = 0.004). Patients with BRAF-specific antibodies are likely to have increased ESRs compared to those without these antibodies. Although the ESR is a non-specific marker of inflammation, ESR values are indeed positively correlated with severe inflammation. On the other hand, patients with prolonged disease in our study cohort had significantly higher levels of anti-BRAF antibodies (18/62) than patients with recent onset disease (2/35) (p = 0.006). With respect to disease status, anti-P25-positive patients had a significantly higher risk of incurring active disease than anti-P25-negative patients (p = 0.034). However, there was no significant difference in the anti-BRAF status among patients with active disease (p = 0.704). This indicates that anti-P25 is more closely correlated with RA than anti-BRAF. The ability of anti-BRAF to activate BRAF, thus activating the MAPK pathway, may be an appropriate explanation for the associations between anti-BRAF and variables of inflammation or disease activity in RA. Charpin, et al. proposed a model to explain how extracellular autoantibodies to BRAF may activate intracellular BRAF [10]. In their model, autoantibodies to BRAF enter the cells as immune complexes via cellular uptake. It is suggested that soluble IgG immune complexes might undergo degradation after uptake [22]. However, it remains unclear how immune complexes formed by BRAF and anti-BRAF antibodies resist degradation from intracellular proteinases.

A limitation of the current study is the inability to collect additional information regarding ESR and other demographic data for SLE and pSS patients who participated in this research as the disease controls, which left us unable to explore the correlation between BRAF-specific antibodies and ESRs for each patient. Further evaluation of BRAF-specific antibodies in autoimmune diseases and other inflammatory diseases would strengthen the conclusions of this study.

In summary, we have observed a similar prevalence of autoantibodies to the intact catalytic domain of wild-type BRAF and a peptide derived from this domain in patients with RA, pSS, and SLE. The associations of anti-BRAF and anti-P25 with disease variables of RA suggest that BRAF-specific antibodies may participate in the inflammatory responses involved in RA. Our conclusion is that anti-BRAF catalytic domain antibodies and anti-P25 antibodies are not specific markers for RA, but the higher titers of BRAF-specific antibodies may be associated with increased inflammation in RA. This finding is contradictory to that of previous studies. The results presented here contribute to our understanding of the pathogenesis of RA and provide insights into the development of potential intervention targets for repressing inflammation. Extensive studies on antibody responses to BRAF in other autoimmune diseases such as pSS and SLE might contribute to a comprehensive understanding of its role in autoimmune disorders.
